# Virtual Quartz Crystal Microbalance: Bioinspired Resonant Frequency Tracking

**DOI:** 10.3390/biomimetics7040156

**Published:** 2022-10-08

**Authors:** Ioan Burda

**Affiliations:** Physics Department, Babes-Bolyai University, 400084 Cluj-Napoca, Romania; ioan.burda@ubbcluj.ro

**Keywords:** QCM sensor, bioinspired technology, virtual impedance analyzer, Allan deviation, virtual QCM, drive level dependence, quartz crystal nonlinearity

## Abstract

The reference acoustic properties of the quartz crystal used as a resonator are ensured by its high-quality factor (Q-factor). The microbalance of the quartz crystal (QCM) exploits the bulk acoustic properties of the quartz crystal. Turning a network analyzer or impedance analyzer into a QCM with a decent measurement rate is a challenge. The use of a virtual instrument to implement an impedance analyzer (VIA) provides greater flexibility to the virtual QCM. In this paper, VIA’s flexibility is exploited for the experimental evaluation of conventional scanning procedures and the influence of associated parameters, in order to identify elements that can lead to a limitation of the performance of a virtual QCM. The results of the experimental investigation justify the use of an innovative and optimized bioinspired scanning procedure to effectively track the serial resonance frequency of the QCM sensor. Variable-resolution spatial sampling of the human retina and the ability of the eye to refocus on the area of interest is the source of bioinspiration for achieving an adaptive virtual QCM. The design methodology and physics are described in detail, and the experimental investigations demonstrate the effectiveness of the proposed bioinspired scanning procedure.

## 1. Introduction

The corolla spider (Ariadna) of the Namib Desert [1] selects small quartz crystal pebbles according to their piezoelectric properties and places them in a circle around its entry into the burrow. To these pebbles of quartz crystal, the corolla spider attaches individual silk threads [2,3], building a piezoelectric array sensor that extends its kinesphere in which the spider can locate its prey. The piezoelectric properties of quartz crystals were explored much later [4] by Homo sapiens. The first applications of the piezoelectric phenomenon appeared during the First World War [5] in the form of strategic technology used to detect submarines. The lattice defects in mineral quartz crystals have long been exploited for age determination, as geochronometers [6].

The use of a quartz crystal as a resonator benefits from its high-quality factor (Q-factor), responsible for its reference bulk acoustic properties. They ensure excellent short-term stability of its series resonance frequency, without having another equivalent among electronic devices. The Q-factor of the resonator has been exploited over a wide range of applications in the field of radio communications [7], time and frequency references [8], or as radio frequency filters [9]. In addition, the resonance frequency dependence on the: temperature [10], mechanical vibration [11], and drive level [12] should be mentioned. Through a design that is specific to each application, these behaviors are avoided or kept under control.

The series resonance frequency of the quartz crystal, the QCM sensor, is changed by adding or removing small amounts of mass from the electrode surface. The quartz crystal microbalance (QCM) exploits the bulk acoustic properties of quartz crystals, and is initially used under the specific conditions of the gaseous medium [13]. By monitoring series resonant frequency changes, real-time information is obtained regarding the molecular interactions or reactions that take place on the QCM sensor surface. With the demonstration of its capability to operate in a liquid medium [14,15] its use as a label-free chemosensors or biosensor was opened. Initially, the measurement of the resonance frequency of the QCM sensor was based on an active method, an oscillator, with only the linear dependence between the series resonant frequency and the mass deposited on the electrodes being of interest. The decrease in the Q-factor due to the dissipation process specific to the liquid medium led to the evolution of the oscillator circuit [16,17] or the development of passive measurement methods [18]. The representative passive methods were taken from the electrical circuits network analysis being materialized in the form of the network analyzer or the impedance analyzer. These perfect methods from the perspective of the revealed electrical properties were bypassed in a first stage because of the costs associated with such professional grade equipment, the measurement time, or due to the low resolution in relation to the Q-factor of the QCM sensor. These historical technological limitations have led to the development of simplified passive methods adapted for measuring the key electrical properties of the QCM sensor. In this framework was developed an extremely elegant passive method known as microbalance crystal quartz with dissipation (QCM-D) that allows the simultaneous measurement of the series resonance frequency and the dissipation factor, and in this way, the viscoelastic and conformational properties of the sample [19,20,21,22] are also monitored.

The reconstruction of the initial function in continuous time from the samples implied compliance with the sampling theorem [23]. The general-purpose methods used to measure the parameters of the QCM sensor cannot comply with this condition in all circumstances. Fortunately, the biological processes taking place on the surface of the QCM sensor are usually very slow [24].

Several dedicated methods can measure the dynamics of rapid processes occurring on the surface of the QCM, such as the evaporation of a microdroplet from a high volatility sample [25]. In this case, the oscillator-based method has no rival if the information provided by it is processed using analog frequency–voltage converters [26,27]. Regarding the ability to track the dynamics of interactions on the QCM sensor surface, the QCM-D version based on the ring-down method [20] is fast enough to cover most of the situations encountered in applications.

The measurement of the four electrical parameters of the Butterworth van Dyke (BVD) lumped elements [28,29] is beneficial in many situations for understanding the complexity of the interactions taking place on the QCM sensor surface. The methods developed for this purpose are based on the network analyzer [30] or impedance analyzer [31], and can successfully approach the measurement of electrical parameters of the BVD model lumped elements. In QCM based on impedance analyzer, the impedance or admittance of the QCM sensor is measured to accurately determine the series resonance frequency and bandwidth [32,33]. No other method of measuring the electrical parameters of the QCM sensor following the BVD model can achieve their accuracy and refinement [34]. In conventional impedance spectroscopy, the passive interrogation frequency is changed with constant steps over the frequency range covering the resonance peaks. The method has a significant limitation relative to the ability to track the dynamics of interactions on the QCM sensor surface, due to the slow acquisition rate. The low acquisition rate should not be understood only as a technological limitation, but rather as a physical limitation given by the steady state condition to be met at the moment of measurement [35]. From this point of view, turning a network analyzer or impedance analyzer into a virtual QCM with a decent measurement rate is a challenge [36].

The use of a virtual instrument to achieve an impedance analyzer (VIA) brings the advantage of software implementation, ensuring greater flexibility [31]. In this paper, this facility is exploited for the experimental evaluation of the conventional scanning method and the influence of the associated parameters, respectively, and their optimization, to avoid limiting the performance of the virtual QCM. The results of these experimental investigations ensure the understanding of the effects produced by the conventional scanning procedure and associated parameters, and based on these measurements, an innovative bioinspired scanning procedure to track the series resonance frequency is proposed and investigated in detail. Bioinspired applications [37] use existing technologies and processes to achieve a performance that is beyond traditional approaches. The bioinspiration source for tracking the series resonance frequency of the QCM sensor is the spatial sampling with a variable resolution of the human retina and the ability of the eye to refocus on the area of interest. This paper makes the following contributions: (i) the investigation of the effects produced by the conventional scanning procedure, and the influence of the associated parameters, (ii) an innovative bioinspired scanning procedure for virtual QCM, and (iii) an experimental evaluation of the performance obtained through this approach.

This work is organized as follows: Section 2 describes the QCM sensor and the principle of operation of a virtual QCM, and Section 3 presents the effects of the conventional scanning procedure and associated parameters proposing the bioinspired tracking of the series resonance frequency, respectively, and its experimental validation. The next two sections are dedicated to a discussion on the benefits of the bioinspired approach and conclusions.

## 2. Materials and Methods

### 2.1. Virtual Impedance Analyzer and BVD Model of the QCM Sensor

**Figure 1:**
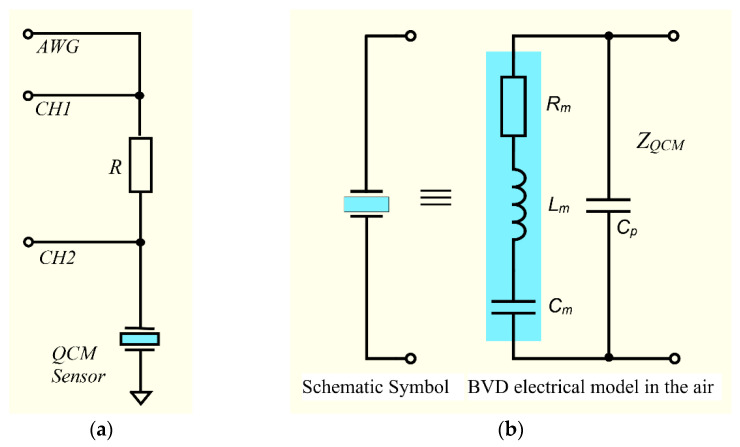
Virtual QCM: (**a**) Front-end circuit with QCM sensor connected to the ground, (**b**) Butterworth van Dyke (BVD) model of the quartz crystal used as QCM sensor.

In recent decades, advanced virtual instruments based on Field Programmable Gate Array (FPGA) circuits are increasingly present. These instruments provide user-configurable analog and digital input–output channels, and any traditional instrument can be implemented, but equally, extremely compact and high-performance dedicated instruments can be imagined. The minimum hardware extension of a virtual instrument to be transformed into VIA consists of an extremely simple front-end circuit shown in Figure 1a.

The equivalent electrical circuit that models the piezoelectric properties of a bulk acoustic resonator used as a QCM sensor is known as the BVD model, and consists of a series arm containing the motional elements $R_{m}$, $L_{m}$, $C_{m}$ in parallel, with a static capacitance $C_{p}$ given by the electrodes. The BVD model shown in Figure 1b contains the lumped electrical elements that must be measured directly or calculated [38]. The virtual QCM approach involved tracking and recording the changes induced by interactions that occur on the surface of the QCM sensor during the experiment. A passive method of measuring the impedance of the QCM sensor based on half-bridge in a commercially available front-end circuit configuration has been proposed and investigated [31]. In the configuration investigated in this paper, an electrode of the QCM sensor is connected to the ground, thus offering the possibility of simultaneous electrochemical measurements [39]. Choosing a reference resistor *R =* 1 KΩ and a careful calibration of VIA ensures a wide range of impedance measurements.

The impedance of the QCM sensor is measured by sweeping the sine wave frequency around its resonant frequencies [31]. Based on a direct digital synthesis algorithm, the virtual instrument implements the arbitrary waveforms generator (AWG) responsible for the sine wave. The impedance value of the QCM sensor is given, in terms of voltage, by:

(1) $$Z=R\left(\frac{V_{CH2}}{V_{CH1}-V_{CH2}}\right)=R\frac{V_{CH2}}{V_{R}}$$

where, $V_{R}$ is the voltage across the reference resistor (*R*). The very high impedance of the analog input channels allows the use of this simplified configuration, Figure 1a, to measure the impedance of the QCM sensor in the air. Without this high impedance of the analog input channels such a configuration is not recommended.

The virtual instrument chosen for the measurement of the electrical parameters of the QCM sensor is the Analog Discovery 2 (AD2) from Digilent Inc., Pullman, WA, USA [40,41]. The virtual instrument controls the measurement sequence specific to the VIA, acquires the data, and calculates the BVD parameters of the QCM sensor in real-time. The AD2 is a virtual instrument containing two 14-bit (100 MSPS) ADCs, two 14-bit (100 MSPS) DACs, and 16 bidirectional digitals I/Os, around a Xilinx Spartan 6 (XC6SLX16-1L) FPGA. The maximum voltage at analog input channels is ±25 V, with an absolute resolution of 0.32 mV for a scale of 5 V or less. The voltage range of the output channels of the AWGs is ±5 V. The input impedance of the analog channels is 1 MΩ versus 24 pF in parallel.

**Figure 2:**
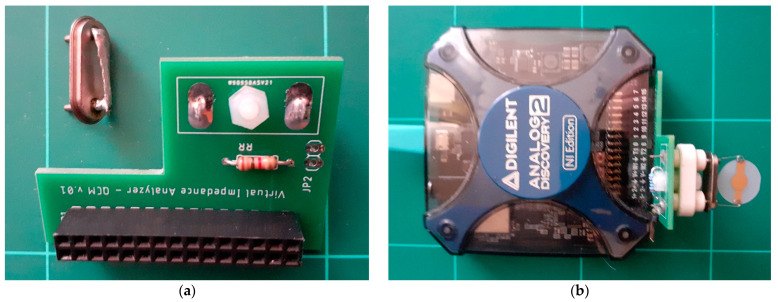
The virtual QCM: (**a**) half-bridge circuit in shunt configuration and the support device for short circuit compensation, (**b**) experimental setup of the virtual QCM.

The VIA circuit shown in Figure 2a was designed as a shield of Analog Discovery 2. In this way, the circuit from Figure 1a is integrated with the data acquisition and is under software control. The experimental configuration is complemented by the QCM flow cell kit (011121, ALS Co., Ltd., Tokyo, Japan) to allow the measurements to occur in a liquid medium [31].

The WaveForms software development kit (SDK) provides access to a public application programming interface (API) that gives users the ability to create custom applications [40]. The software applications (app) written in the Python language exploit the SDK functions and provide support for the experimental investigation of the effects induced by the scanning procedures and parameters, or the experimental validation of the concept of virtual QCM. An analog output channel is used for passive interrogation with a sine wave of the QCM sensor. For this procedure, the SDK has dedicated functions, thus ensuring an efficient approach to the software application. The PC-type host computer controls through the USB interface the settings of the virtual QCM, ensures the processing of the measured signals, calculates the BVD lumped electrical parameters for the QCM sensor, and ensures the results are displayed in real-time.

### 2.2. Impedance Spectroscopy of the QCM Sensor

As seen in Figure 1b, the BVD model implies the existence of two arms in parallel. One of the two arms is called static and contains only a capacitance $C_{p}$ determined by the existence of QCM sensor electrodes. The second arm models the piezoelectric properties of the QCM sensor [34]. This arm consists of two complex conjugated circuit elements that model the energy storage process, and a third element that models the dissipative processes of the QCM sensor. The impedance of the QCM sensor is given by the relation:

(2) $$Z_{QCM}=\frac{Z_{p}Z_{m}}{Z_{p}+Z_{m}}.$$

where $Z_{p}$ and $Z_{m}$ are the impedance of the static and motional arm. At the frequency $F_{r}$, called the series resonance frequency, the motional arm reactance is zero and it can be calculated with the following equation:

(3) $$F_{r}=\frac{1}{2\pi \sqrt{L_{m}C_{m}}}.$$

Due to the presence of parallel capacitance, the QCM sensor also has an anti-resonance frequency $F_{ar}$ that is given by:

(4) $$F_{ar}=F_{r}\sqrt{1+\frac{C_{m}}{C_{p}}}.$$

The parallel or anti-resonance frequency is always after the series resonance frequency, and their separation is determined by the ratio of the capacitances $C_{m}$ and $C_{p}$. The direct measurement or calculation of the parameters of the electrical elements of the BVD model provides a detailed description of the interactions that change the mass and viscosity, or highlight the electrical properties of the analyte molecules attached to its surface. It is easy to demonstrate the capability of a VIA-based QCM in relation to a QCM-D, given the Q-factor expressed by the BVD lumped elements:

(5) $$Q=\frac{1}{2\pi F_{r}R_{m}C_{m}}=\frac{2\pi F_{r}L_{m}}{R_{m}}=\frac{1}{D}$$

In an equivalent transcript, we can consider the following equations:

(6) $$D=\frac{R_{m}}{2\pi F_{r}L_{m}}=2\pi F_{r}R_{m}C_{m}=\frac{1}{Q}$$

(7) $$R_{m}=\frac{D}{2\pi F_{r}C_{m}}=D2\pi F_{r}L_{m}$$

In the VIA method, Figure 1a, the interrogation sine wave from *AWG* is applied to analog input channel CH1, and through a reference resistor *R* to the input channel CH2 and to the QCM sensor. The data acquisition for both analog input channels is digitally synchronized, thus making it possible to measure the impedance of the QCM sensor and its phase response.

The first electrical parameter of the BVD model for the QCM sensor to be determined is the shunt and stray capacitance $C_{p}$, by measuring the impedance at a frequency that is much lower than its resonance frequencies. For example, considering a particular case, the measurement of the shunt and stray capacitance for a QCM sensor with a series resonance frequency of 10 MHz is carried out at a frequency of 1 MHz. The computation of the shunt and stray capacitance is performed based on the following relation:

(8) $$C_{p}=-j\frac{1}{2\pi F_{m}Z_{p}}$$

where, $F_{m}$ is 1 MHz and $Z_{p}$ is the impedance measured at this frequency.

A second measurement aims to determine the motional capacitance $C_{m}$ of the QCM sensor in the air. This involves measuring the impedance of the QCM sensor in the range of resonant frequencies. First, phase data are investigated using the software, to confirm the capacitive–inductive–capacitive transition of the reactance within the chosen frequency scanning range. If the scanning range is wrong, based on phase information, a new set of the frequency start and frequency stop is updated and the impedance measurement is reloaded. By applying the methods described in the literature [31,34], the electrical elements parameters of the BVD model are determined. The value of the motional capacitance $C_{m}$ is retained in this stage and it can be expressed in the form:

(9) $$C_{m}=C_{p}\left({\left(\frac{F_{ar}}{F_{r}}\right)}^{2}-1\right)$$

where, $F_{ar}$ and $F_{r}$ are directly correlated with the maximum and minimum of the raw data measured using VIA. The value of the shunt and stray capacitance $C_{p}$ and motional capacitance $C_{m}$ measured in the air are retained and used by the virtual QCM algorithm.

From this point on, the transition to the operating algorithm specific to a virtual QCM is completed. In a commercial configuration of the front-end circuit used by the virtual instrument AD2 for VIA application, experimental investigations were carried out, and computational algorithms were developed [31,34]. A VIA is not a QCM, because a QCM must have the ability to track interactions on the surface of the QCM sensor to record and display them throughout the experiment. Converting an impedance analyzer into QCM must ultimately ensure a decent measurement rate. The virtual QCM’s measurement rate depends on the scanned frequency range and the scanning step. The ability to adapt virtual QCM to experimental reality depends on the scanned frequency range, and the accuracy of the measurements depends on the scanning step. The challenge is to find an innovative adaptive scanning procedure without compromise for scanning range, or for the accuracy of the measurement of the series resonance frequency. On the other hand, this scanning procedure must ensure the above-mentioned performance by using minimal hardware resources that allow for the design of a portable and inexpensive QCM.

In virtual QCM, scanning takes place only around the series resonant frequency by monitoring its change. For a limited range of the acquired data around series resonance frequency, they are fitted with the impedance of the motional arm, Figure 1b, expressed by the relation:

(10) $$Z_{m}\left(f\right)=R_{m}+j\left(2\pi fL_{m}-\frac{1}{2\pi fC_{m}}\right).$$

The value of the motional capacitance is previously determined using a fitting procedure, and the value of motional inductance and resistance is obtained. The following series resonant frequency is calculated based on Equation (3). The series resonant frequency is subject to the tracking procedure to monitor the interactions taking place on the surface of the QCM sensor. Motional resistance is preferred in the case of virtual QCM to illustrate dissipative processes, instead of the Q-factor or dissipation expressed by Equations (5) and (6). These parameters are calculated using the VIA component of the virtual QCM, and their values are known during the measurements without being displayed as a time function. The determination of the electrical parameters of the BVD model elements is a major advantage brought by the virtual QCM from the perspective of the monitoring capability of the interactions that takes place on the surface of the QCM sensor.

### 2.3. Compensation of the Virtual Impedance Analyzer

**Figure 3:**
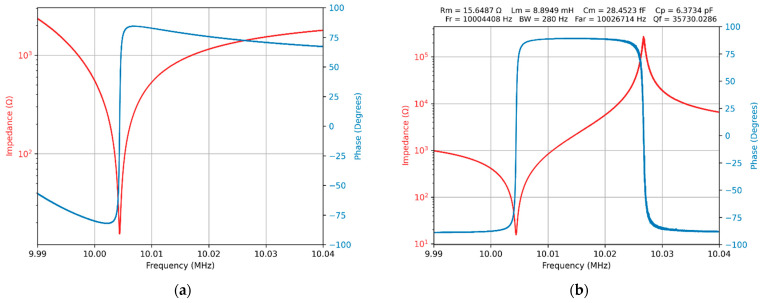
The virtual impedance analyzer (VIA): (**a**) QCM sensor in the air without compensation, (**b**) QCM sensor in the air with open- and short-circuit compensation.

Impedance compensation methods are extensively described in the literature [31,42] for AD2, and are based on the so-called nulling method [43]. This method performs at least one of the following processes: (i) open-circuit compensation to compensate for the open-circuit stray impedance $Z_{oc}$, and (ii) short-circuit compensation to compensate for the short-circuit stray impedance $Z_{sc}$. Once $Z_{oc}$ and $Z_{sc}$ have been measured, the compensated impedance of the QCM sensor $Z_{Q}$ is calculated as follows:

(11) $$Z_{Q}=\frac{Z_{rm}-Z_{sc}}{1-\left(Z_{rm}-Z_{sc}\right)/Z_{oc}}$$

where $Z_{rm}$ is the QCM sensor raw measured impedance. The effect of the compensation procedure is shown in Figure 3.

The open- and short-circuit compensations are supported by SDK functions and can be performed using the WaveForms app or dedicated apps inspired by support examples. The frequency sweep range for the QCM sensor is very narrow, so the compensation procedure can be reduced to four constants. They are resistance and reactance for each of the two boundary situations mentioned above. The open-circuit compensation is used to compensate for the stray capacitance. Short-circuit compensation, in addition to open-circuit compensation, is needed for QCM sensors with a very low impedance. It should be remembered that for an impedance analyzer the most difficult task is to perform measurements in a wide impedance range, as is the case for the unloaded QCM sensor in the air. For any other method of measuring the parameters of the QCM sensor, the opposite situation is encountered. The results illustrated in Figure 3b confirm the ability of the front-end circuit presented in Figure 1a to accurately measure the impedance of the QCM sensor in air.

### 2.4. Allan Deviation Analysis

**Figure 4:**
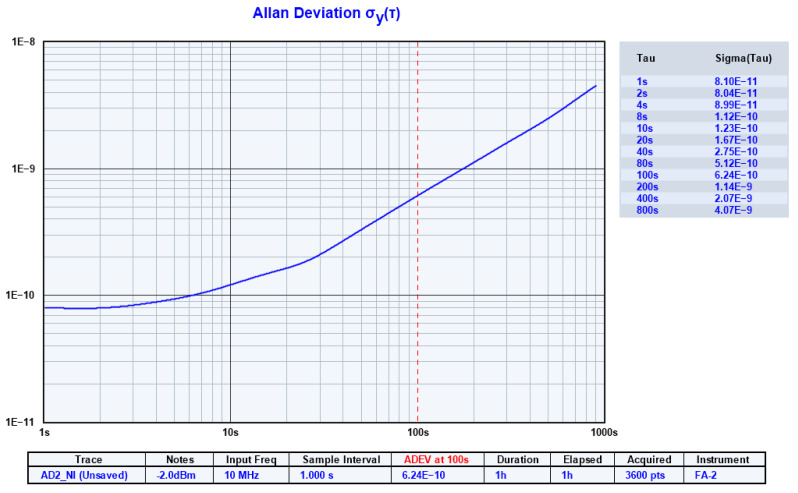
Allan deviation of the arbitrary wave generator (AWG) of the AD2.

Due to the long measurement time for virtual QCM-based biosensor applications, a justified question is related to the frequency stability of the AWG implemented with the virtual instrument AD2 [24]. The most well-known measurement of the stability of an oscillator is the Allan deviation [44,45]. The Allan Deviation is a unitless measure of stability that is used to quantify the stability of clocks or frequency reference sources. The Allan deviation for the AWG set to generate a 10 MHz sine wave is shown in Figure 4.

An Allan deviation of 6.24 × 10^−10^ at a time of observation 100 s should be interpreted as instability in frequency between two observations, distanced to 100 s, with a relative mean root square value (RMS) of 6.24 × 10^−10^. For a reference frequency of 10 MHz, this would be equivalent to an RMS change of 6.24 mHz. The Allan deviation analysis shows a performance beyond the expectations of the virtual instrument AD2 from the perspective of its stability.

## 3. Results

In this section, a coherent approach to the process of transforming VIA into virtual QCM is supported by a deep investigation of the effects produced by the conventional scanning method, and the influence of the associated parameters. The uncertainty of raw data disrupts the fitting algorithm, inducing a significant decrease in the resolution of the virtual QCM. After an experimental investigation considering the parameters that can negatively influence the performance of the virtual QCM, an optimal bioinspired scanning procedure is proposed from the perspective of the frequency tracking range, accuracy, and measurement rate. Finally, an experimental investigation validates the virtual QCM implemented based on the bioinspired tracking of the series resonance frequency. A QCM sensor with a series resonant frequency of 10 MHz (151225-10, International Crystal Manufacturing Co., Inc., Oklahoma City, OK, USA) was used. During the experiments, the temperature in the laboratory was 21 ± 2 °C, with a relative humidity of 50 ± 10%.

### 3.1. Series Resonance Frequency Tracking Range

**Figure 5:**
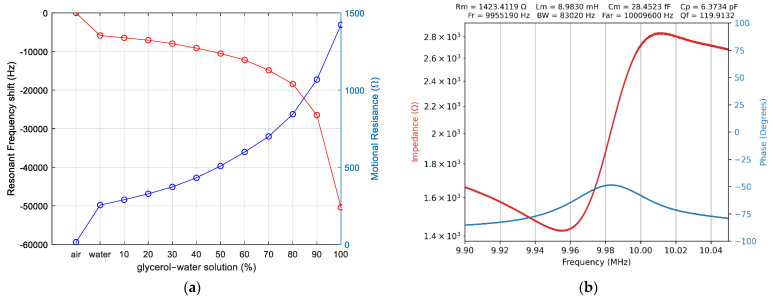
The virtual impedance analyzer (VIA): (**a**) series frequency resonance shift and motional resistance from air, water, and glycerin–water solution, (**b**) QCM sensor in 100% glycerin.

For a virtual QCM, an adaptive concept is mandatory to be able to cover a wide range of experimental situations. In this regard, a first experimental investigation aims to determine the variation range of the series resonance frequency and motional resistance when the surface of the QCM sensor passes from air to water and a decadic glycerin–water solution [46]. The results of these experimental investigations are shown in Figure 5.

Figure 5b confirms the VIA performance under heavy load conditions, when one of the electrodes of the QCM sensor is 100% glycerin. Based on these investigations, it can be concluded that a scanning range of 5000 Hz centered on the series resonance frequency ensures the successful tracking of changes that occur, in most experimental situations.

### 3.2. Scanning Step Effect

**Figure 6:**
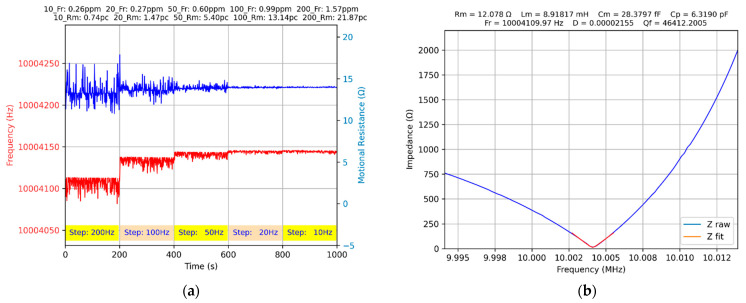
The QCM sensor measured in air: (**a**) accuracy of series resonant frequency (ppm) and motional resistance (%), (**b**) series resonant frequency tracking with constant step equal to 200 Hz.

To determine the effect of the scanning step, a dedicated Python app was written. The scanning step is changed every 200 s according to the algorithm shown in Figure 6a.

In the VIA component of Python app shown in Figure 6b, the raw data acquired around the series resonance frequency are illustrated, and their fit with Equation (10).

In 450 ms, the Python app ensures the acquisition of 100 samples and their transfer to the PC. The fitting procedure, in a narrow range around the minimum impedance of the QCM sensor, with Equation (10), takes no more than 2 ms, and the value of the impedance, $L_{m}$ and motional resistance, $R_{m}$ is determined. The remaining time up to the next measurement is used to update the display. After each measurement cycle, the series resonant frequency is calculated on the basis of Equation (3), and around this frequency, another 100 samples are acquired. For every 200 s, a scanning step is calculated in parts per million (ppm), and the measurement accuracy of the series resonance frequency and of the motional resistance expressed in percent (pc, %). The results of these experimental investigations are summarized in Table 1.

The accurate determination of the series resonant frequency depends significantly on the scanning step. A step of 10 Hz is perfectly relative to the stability of the AD2 virtual instrument proven in Section 2.4.

### 3.3. Drive Level Dependence

**Figure 7:**
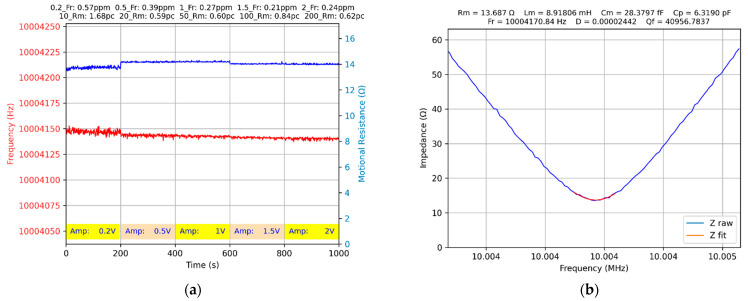
The QCM sensor measured in air with 10 Hz step: (**a**) drive level dependence, (**b**) series resonant frequency tracking with 0.2 V sine wave amplitude.

The drive level dependence (DLD) of the quartz crystal behavior is well known in the literature [12]. Based on this information, an experimental investigation of DLD in the case of the proposed experimental setup in Figure 1a is mandatory. A dedicated Python app has been written, and every 200 s, the amplitude of the interrogation sine wave is changed according to the algorithm shown in Figure 7.

In the VIA component of the virtual QCM shown in Figure 7b, a typical situation is presented in the case of the interrogation sine wave with an amplitude of 0.2 V. For an amplitude of 0.2 V, the response of the QCM sensor is affected by noise, reducing the measurement accuracy of the series resonant frequency and the motional resistance. The results summarized in Table 2 confirm the existence of a DLD effect that cannot be ignored in VIA measurements.

### 3.4. Scanning Direction Effect

**Figure 8:**
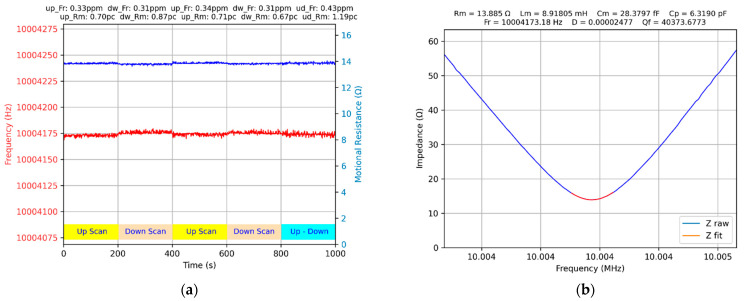
The measurement of the QCM in air with 10 Hz scanning step and 2 V sine wave amplitude: (**a**) scanning direction effect, (**b**) series resonant frequency tracking.

The existence of a slight nonlinearity effect of the QCM sensor response is investigated experimentally by controlling the scanning direction [47]. For carrying out this experimental investigation, a dedicated Python app was written. The scanning direction is changed every 200 s. The procedure involves a scanning direction from low to high frequencies (Up Scan) or from high frequencies to low frequencies (Down Scan), as shown in Figure 8a. In the time interval from 800 to 1000 s, the scanning direction is interlaced (Up–Down), and this procedure causes an increase in the uncertainty of measuring the series resonance frequency of the QCM sensor. The average frequency shift due to the scanning direction is only 0.23 ppm.

As previously mentioned, this effect is not significant, and practically does not influence the usefulness of the information provided by a QCM, based on a passive scanning procedure. In the case of the QCM method, only the relative frequency change is evaluated; an error in measuring the absolute value of the frequency is not relevant. At this time, the presence of this effect, which is typical of nonlinear systems, is demonstrated [47]. This effect is present under ideal conditions, with the QCM sensor in the air without any sensing layer. The study of DLD or the nonlinear response of the quartz crystal is typical for its applications in time or frequency references sources [12]. These phenomena occur sporadically and depend on the manufacturing technology of the QCM sensor, on its exposure to high levels of excitation, or can be determined by the aging process.

### 3.5. Bioinspired Series Resonant Frequency Tracking

**Figure 9:**
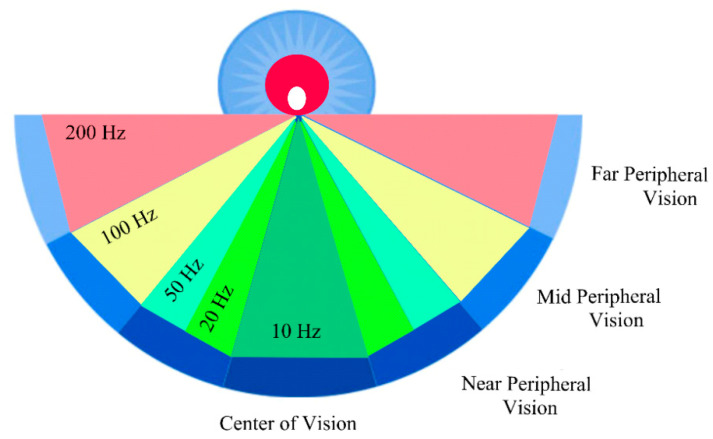
The basic concept of bioinspired series resonant frequency tracking.

Bioinspired sensors use existing technologies and processes to achieve performances that are comparable to their natural counterparts. The biological visual system is frequently a source of inspiration in computer vision [48]. The source of bioinspiration for the series resonant frequency tracking of the QCM sensor is the nonuniform sampling of the human retina and the ability of the eye to focus on the interest zone. The macula lutea [49] is responsible for central vision; it consists of two parts: the fovea with high acuity and the parafovea with a lower spatial resolution. Beyond the macula, a rapid decrease in the spatial resolution is specific to peripheral vision; in turn, it is divided into several zones, as shown in Figure 9.

In addition, an attention algorithm inspired by eye movement is implemented to track the series resonant frequency of the OCM sensor. The frequency in the center of the high-resolution scanning range is the frequency at which the QCM sensor impedance was minimal in the previous scanning cycle. Based on the investigations in Section 3.1, the range of scanning frequencies can be reduced to approximately 5000 Hz to maintain an adaptive capability of the virtual QCM for most experimental situations. To make a measurement per second, only 100 samples can be purchased in the particular case of virtual QCM based on AD2.

**Figure 10:**
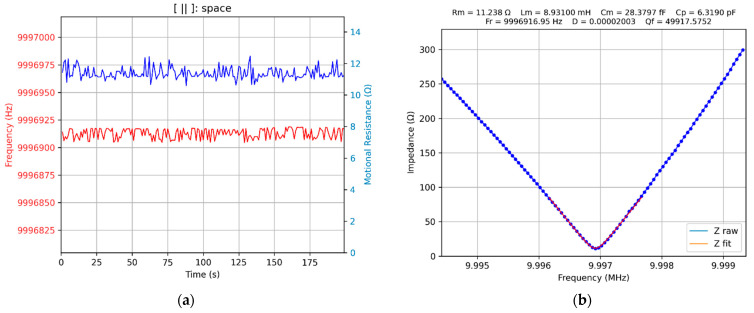
The virtual QCM: (**a**) series resonance ($F_{r}$) and motional resistance ($R_{m}$), (**b**) series resonant frequency tracking with 50 Hz constant steps in the VIA.

To compare the effects produced using the conventional and bioinspired scanning procedures, a Python app was written, which in the first 200 s determines the series resonance frequency of the QCM sensor based on the acquisition of 100 equidistant samples in the next 200 s based on 100 samples distributed according to Table 3. In the conventional scanning procedure with a constant step of 50 Hz (equidistant samples) the scanned frequency range is 5000 Hz, and in the case of bioinspired, the scanned frequency range is 5800 Hz. The fitting algorithm is identical in both situations, and the result of the constant step scan is shown in Figure 10.

**Figure 11:**
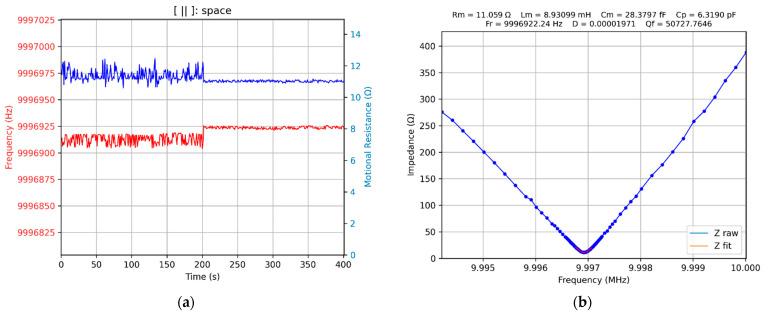
The virtual QCM: (**a**) series resonance ($F_{r}$) and motional resistance ($R_{m}$) with constant steps (50 Hz, 0–200 s) and bioinspired steps (200–400 s), (**b**) bioinspired series resonance frequency tracking in VIA.

The experimental results obtained in the case of bioinspired scans are shown in Figure 11. A significant decrease in uncertainty in the measurement of the series resonant frequency and motional resistance can be observed. This result has already been experimentally confirmed in Section 3.2.

After completing the investigations described above, it was possible to write a Python application that allows for the extension of a VIA with the virtual QCM component. The VIA component is available in real-time, as can be seen in Figure 10b or Figure 11b. The virtual QCM is a VIA extension with the possibility of bioinspired tracking of the series resonant frequency. Finally, from the perspective of the measurement rate, an efficient virtual QCM was created, making it possible to use a VIA as electronic support for a biosensor. Considering the same scanning range around the series resonant frequency, the advantage of the bioinspired scanning procedure over the conventional procedure is clearly highlighted in Figure 11.

### 3.6. Evaporation of an Isopropyl Alcohol Drop

**Figure 12:**
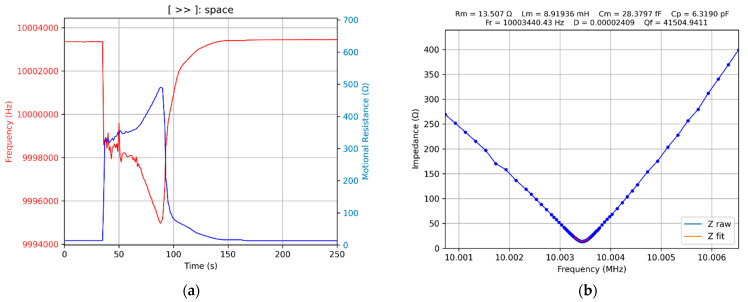
Isopropyl alcohol: (**a**) drop contact with the surface of the QCM sensor, followed by spread and evaporation, (**b**) bioinspired tracking of the series resonance frequency.

Experimental validation of the bioinspired procedure for tracking the series resonance frequency of the QCM sensor was carried out by adding an isopropyl alcohol drop onto one of the electrodes. The result of this experimental investigation is shown in Figure 12.

The transition from the air to the liquid medium is well highlighted, followed by the evaporation of the isopropyl alcohol droplet. In addition, the process of expansion of the isopropyl alcohol droplet on the surface of the electrode of the QCM sensor is well monitored. The existence of a residue in the isopropyl alcohol is easy to observe after stabilizing the series resonant frequency at the end of the evaporation process. As seen in Figure 12 of the final Python app, virtual QCM is implemented as a VIA extension, and during the experiment, the specific information for an impedance analyzer is also present (Figure 12b).

## 4. Discussion

Vision is the most complex and useful of the senses for humans [49]. A significant part of the cerebral cortex is involved in the processing of visual information that is obtained from more than 50% of the sensory receptors in the human body, located in the eye. Due to the complexity and elegance of highly efficient solutions produced by nature in relation to a limited processing resource, it is a source of inspiration for many applications [48]. A bioinspiration approach is useful for ensuring a measurement rate that is equivalent to oscillator-based methods. The oscillator-based conventional method provides a limited picture of the interactions occurring on the surface of the QCM sensor. It should be noted that no method covers every situation; in the literature, several methods cover particular situations [46]. Capitalizing on the resources of a virtual instruments is not limited to the implementation of known methods through a new technology, but also through an innovative reconsideration of them. The original element of the proposed bioinspired scanning procedure is given by the possibility of having a virtual QCM as an extension of a VIA, and not its absorption, specific to conventional approach.

A quartz crystal can exhibit specific behaviors if the drive level is moderately high without being generalized. DLD is determined by technological defects, using an operation outside the technical specification that causes irreversible changes, or via an unfavorable aging process [12,47]. Under normal operating conditions, these effects do not occur, or their manifestation is negligible, as is shown in Figure 8a. In particular, considering passive measurement methods, they can benefit from a higher drive level up to the maximum limit that is recommended by the technical specification of the QCM sensor. In the configuration shown in Figure 1a, the reference resistor, *R =* 1 KΩ, ensures a strong current limitation through the QCM sensor, and this situation significantly reduces DLD or nonlinear effects. Furthermore, the measurement in a liquid medium increases the value of the motional resistance, and this modification is in the favor of passive QCM methods based on the impedance analyzer. The above findings are relevant to measurement techniques that are explicitly based on the impedance spectrum characteristics of the QCM sensor.

## 5. Conclusions

Critical situations induced by the conventional scanning procedure, and the influence of the associated parameters from the perspective of using VIA as a virtual QCM have been investigated experimentally. After evaluating each situation, a bioinspired procedure is finally proposed as the best choice for the effective tracking of the series resonance frequency of the QCM sensor. The effects produced by the drive level and the scanning direction were also investigated. Based on extensive experimental investigations, it was possible to purpose a bioinspired scanning procedure for efficiently transforming a VIA into a virtual QCM. This scanning procedure provides a measurement per second and a wide tracking range of 5800 Hz. The accuracy of the QCM sensor’s series resonance frequency measurement is 0.24 ppm, demonstrated in air without temperature stabilization. The error in measuring the value of motional resistance is 0.68%, and this performance under the experimental conditions described above demonstrates the usefulness of the bioinspired scanning procedure. The bioinspired transformation of a VIA into a virtual QCM simultaneously ensures the information is specific to an impedance analyzer, as well as the monitoring the interactions taking place on the surface of the QCM sensor with a measurement rate that is typical of conventional methods.

## Figures and Tables

**Table 1:** Accuracy of measurement of series resonant frequency (ppm) and motional resistance (%) of the QCM sensor in the air according to the scanning step.

Scanning Step (Hz)	ppm	% (pc)
200	1.57	21.87
100	0.99	13.14
50	0.60	5.40
20	0.27	1.47
10	0.26	0.74

**Table 2:** Accuracy of measurement of frequency (ppm) and motional resistance (%) according to the drive level of the QCM sensor in the air.

Drive Level (V)	ppm	% (pc)
0.2	0.57	1.68
0.5	0.39	0.59
1.0	0.27	0.60
1.5	0.21	0.84
2.0	0.24	0.62

**Table 3:** The scanned frequency range in case of bioinspired resonant frequency tracking.

Scanning Step (Hz)	Steps	Range ^1^ (Hz)
200	2 × 10	4000
100	2 × 5	1000
50	2 × 5	500
20	2 × 5	200
10	50	500

^1^ The total scanned frequency range is 5800 Hz.
